# Changes in olfactory function in children and adolescents with obesity following an inpatient weight loss program: a longitudinal study

**DOI:** 10.1186/s12916-026-05007-3

**Published:** 2026-06-17

**Authors:** Bea Klos, Helene Sauer, Verena Steinhauser, Jessica Godwin, Kathrin Ohla, Valentin A. Schriever, Stephan Zipfel, Paul Enck, Isabelle Mack

**Affiliations:** 1 Department of Psychosomatic Medicine and Psychotherapy, University Medical Hospital, Tübingen, Germany; 2 Science & Research, dsm-firmenich, Satigny, Switzerland; 3 Department of Pediatrics, Division of Pediatric Neurology, Goethe University Frankfurt, Neurometabolics and Prevention, Frankfurt (Main), Germany

**Keywords:** Childhood obesity, Olfaction, Sniffin`stick test, Hyposmia, Normosmia

## Abstract

**Background:**

Obesity is a major global public health burden, yet the mechanisms underlying interindividual variability in treatment response remain poorly understood. Olfactory function influences eating behavior and may differ in individuals with obesity. However, evidence in pediatric populations is limited, and their responsiveness to weight loss remains unclear. This study aimed to assess olfactory function in children and adolescents with obesity before and after an inpatient weight loss intervention. Specifically, we examined whether olfactory performance is within normative values and whether it changes in response to weight reduction.

**Methods:**

Sixty children and adolescents with overweight or obesity (13.0 ± 1.9 years, BMI z-score: 2.5 ± 0.5) underwent a structured inpatient rehabilitation program (mean duration: 38 ± 10 days) integrating nutrition therapy, physical activity, stress management, and behavioral modification. Olfactory function was assessed at baseline (T1, ~ 7 days after admission) and after the intervention (T2, ~ 27 days after T1) using the Sniffin’ Sticks Test©, comprising odor threshold, discrimination, and identification. At both time points, participants self-reported their perceived olfactory ability.

**Results:**

At T1, data were available for 59 participants; 50 completed the follow-up assessment at T2. Completers showed significant reductions in body weight and BMI z-score (*p* < .001). Olfactory function at T1 was largely within the normative range when compared with age-matched reference data. During weight loss, olfactory function remained largely stable: odor identification improved modestly (*p* = .003), whereas odor threshold and discrimination showed no significant changes and were not associated with weight loss. Self-reported olfactory ability showed weak correspondence with objective measures and remained unchanged.

**Conclusions:**

Olfactory function in children and adolescents with obesity was largely within the normative range and showed limited change during short-term inpatient weight loss. These findings suggest that olfactory alterations may not be a primary determinant of short-term treatment response. However, age- and domain-specific variability may still be relevant for how individuals perceive and respond to food-related cues, with potential implications for dietary adherence and long-term weight management. Future studies with normal-weight control groups should examine whether such variability contributes to differences in eating behavior and long-term outcomes.

**Trial registration:**

DRKS00005122.

**Supplementary Information:**

The online version contains supplementary material available at 10.1186/s12916-026-05007-3.

## Background

Obesity constitutes a major global public health burden. According to the World Health Organization (WHO), 59% of adults and approximately 28% of children in the European region are affected by overweight or obesity [1]. Obesity is increasingly understood as a multifactorial condition resulting from the interaction of biological, environmental, and behavioral determinants, ultimately leading to a sustained positive energy balance [1, 2]. In this context, weight loss interventions represent a central strategy for improving obesity-related health outcomes. However, interindividual variability in treatment response remains substantial, and the mechanisms underlying successful weight loss are not yet fully understood.

One factor that may contribute to variability in treatment response is eating behavior, a central determinant of energy intake shaped by sensory inputs including olfaction [3, 4]. Cross-sectional associations between olfactory dysfunction and altered food preferences, reduced food enjoyment, and changes in dietary patterns have been described [5–8], suggesting that olfaction may act as a modulatory factor in eating behavior. However, causal relationships remain to be established, and recent evidence suggests that the impact on actual food choice and intake may be more limited than previously assumed [9].

This is particularly relevant given that olfactory function may itself be altered in individuals with obesity. Cross-sectional evidence from adult populations indicates an association between higher BMI and reduced olfactory function [10, 11], , while some studies report enhanced sensitivity to food-related odors [12, 13]. Whether these associations reflect a causal relationship or are explained by shared metabolic, hormonal, or behavioral factors cannot be determined from the available cross-sectional data. Intervention studies suggest that olfactory function may be dynamic in relation to weight change and metabolic state: improvements in olfactory sensitivity have been reported following bariatric surgery, with a median follow-up of approximately six months [14, 15], though other studies report no significant changes [16, 17].

Whether the relationships between olfactory function and obesity described in adults extend to pediatric populations remains unclear. A systematic review by Cattaneo et al. [18] found no consistent association between obesity and chemosensory function in children, supported by a subsequent study reporting no significant differences in olfactory performance across weight groups [19]. In contrast, other studies report reduced olfactory function in association with obesity-related characteristics [20, 21], or increased sensitivity modulated by pubertal stage [22]. Beyond sensory performance, olfactory cues may differentially influence food choice in children depending on body weight [23], suggesting potential relevance for treatment response.

Currently, treatment of pediatric obesity follows a staged approach broadly similar to that used in adults, from multicomponent lifestyle interventions targeting energy intake, physical activity, and behavioral modification, to pharmacotherapy and bariatric surgery in severe cases [24, 25]. While lifestyle interventions can achieve short-term weight reductions, dropout rates are high and long-term maintenance remains challenging [24], underscoring the need to better understand factors contributing to response variability.

Taken together, these findings highlight a critical gap in understanding the temporal dynamics of olfactory function in pediatric obesity. The reversibility of olfactory alterations in response to weight loss remains poorly characterized in this age group. While adult studies suggest that olfactory function may change following weight reduction, longitudinal data in children, particularly in clinical intervention settings, are scarce. Emerging evidence further indicates that olfactory function may predict treatment outcomes, as higher odor identification performance has been associated with greater weight loss in pediatric obesity interventions [26]. Against this background, the present study aimed to assess olfactory function in children and adolescents with obesity before and after a 27-day inpatient weight loss intervention. Specifically, we examined whether olfactory performance deviates from normative values and whether it changes in response to weight reduction, thereby providing insight into its potential role in explaining interindividual variability in treatment response.

## Methods

### Study design and participants

The study enrolled 60 children (28 males, 32 females; mean age 13.0 ± 1.9 years, range 9–17 years), all classified as overweight or obese (OBE, BMI percentile ≥ 90) and referred for inpatient weight-loss intervention. Participants underwent a structured inpatient rehabilitation program at the Children’s Hospital for Respiratory Diseases, Allergies, and Psychosomatics in Wangen i.A., Germany. The program followed an interdisciplinary approach delivered by a multidisciplinary team of pediatricians, psychologists, dietitians, and physical therapists, comprising group-based nutritional training oriented towards a balanced mixed diet, structured exercise and sports sessions, and psychological support including stress management and behavioral modification strategies. Comprehensive details on eligibility criteria and therapeutic procedures have been published previously [27].

**Fig. 1:**
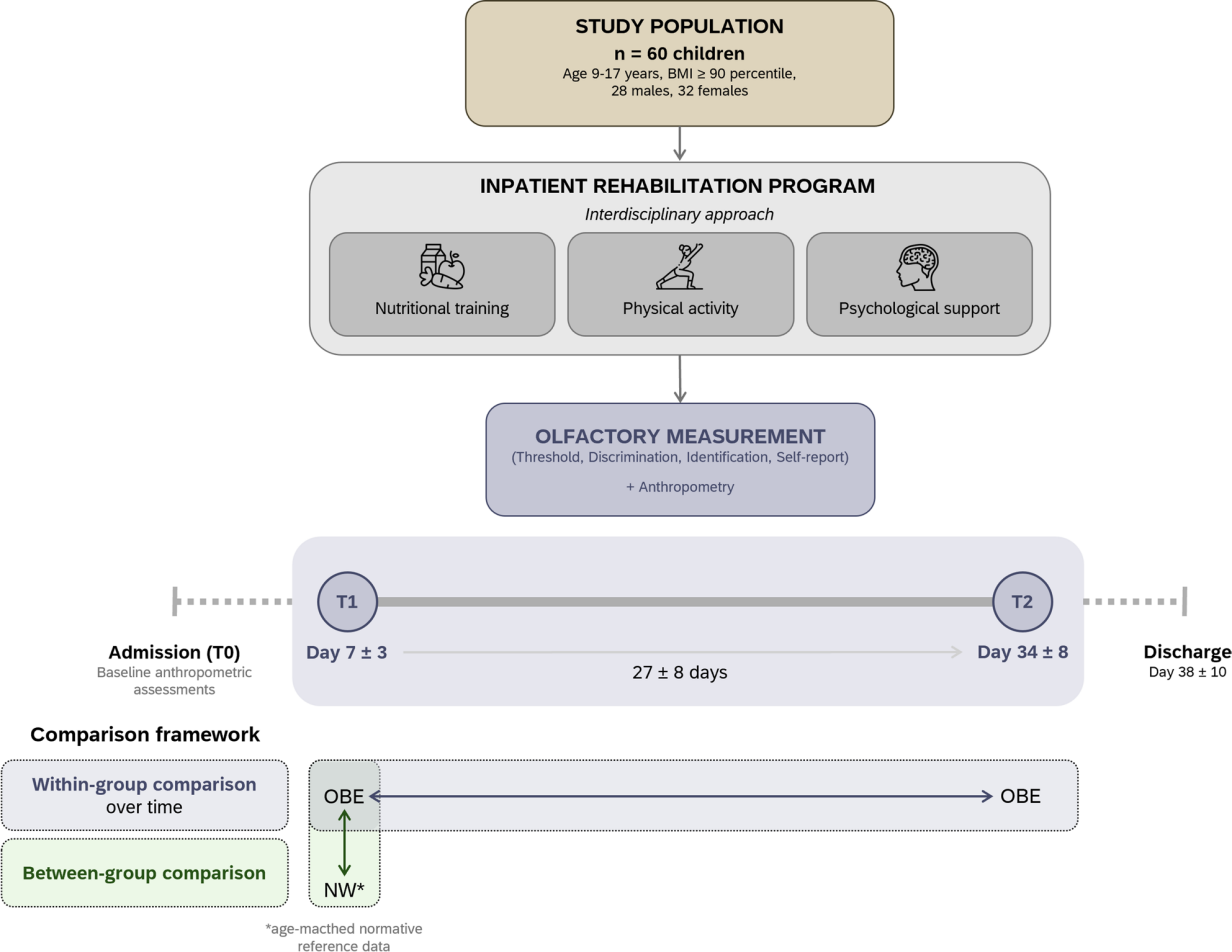
Study design of the olfactory assessment within the DROMLIN study. Study design of the olfactory assessment within the DROMLIN study. Sixty children and adolescents with overweight or obesity (BMI ≥ 90th percentile, age 9–17 years, 28 males, 32 females) underwent a structured inpatient rehabilitation programme (mean 38 ± 10 days) comprising nutritional training, physical activity, and psychological support. Olfactory function was assessed at two time points (T1; T2) using the Sniffin’ Sticks^®^ test battery (threshold, discrimination, identification) alongside self-reported olfactory ability and anthropometric measurements. T0 refers to the routine baseline anthropometric assessment conducted at admission and was not part of the olfactory measurement protocol. T1 olfactory outcomes were compared with published age-specific normative reference data (NW, no control group included): for 9–11 years, Gellrich et al. (2017; *n* = 29 for threshold and discrimination) and Lohrer et al. (2024; *n* = 169 for identification); for ≥ 12 years (2AFC), Gellrich et al. (2017; *n* = 30 for threshold and discrimination) and Lohrer et al. (2024; *n* = 160 for identification); for ≥ 12 years (3AFC), Oleszkiewicz et al. (2019; *n* = 1,750)

The mean duration of hospitalization was 38 ± 10 days (range 16–70). Baseline assessments (T0) were performed by clinical staff during the initial days of inpatient therapy. The first study assessment (T1) was conducted over two consecutive days, on average 7 ± 3 days after admission, allowing participants to acclimatize to the clinical setting and ensuring standardized testing conditions. The second assessment (T2), following the same protocol as T1, was carried out in the final week of inpatient therapy, 27 ± 8 days after T1 (Fig. 1). As no parallel control group was included, olfactory outcomes at T1 (first assessment prior to substantial intervention effects) were compared with age-specific normative data from the literature.

The present work was conducted as part of the DROMLIN study (PreDictor Research in Obesity during Medical Care Weight Loss in Children and Adolescents during Inpatient Rehabilitation) [27], and constitutes a secondary exploratory analysis of olfactory data collected within this study, for which olfactory function was not a primary outcome. Data collection took place between 2012 and 2013. Written informed consent was obtained from both children and their parents after providing detailed information about the study objectives.

### Assessments

#### Anthropometry

Age and sex were obtained from hospital records. Height and weight were measured at all time points using standardized procedures. BMI was calculated and referenced against sex- and age-specific BMI charts to derive BMI percentiles and z-scores at each time point [28]. Percentage body fat was assessed using a Lipometer (Möller Messtechnik, Graz, Austria) at 15 anatomical sites, providing non-invasive and validated measurements [29, 30]. At T1, pubertal status was evaluated using Tanner staging [31, 32] (breast/genital and pubic hair development), with participants self-assigning to the appropriate stage based on standardized reference images. In addition, menarche (girls) and voice change (boys) were recorded as self-reported indicators of pubertal maturation.

#### Olfactory tests

Olfactory testing was conducted in the afternoon, approximately 2.5 hours after lunch, using the Sniffin’ Sticks Test© (Burghart Messtechnik GmbH, Holm, Germany). The Sniffin’ Sticks test employs felt-tip pens filled with odorants and consists of three subtests: odor threshold (T), odor discrimination (D), and odor identification (I). Scores from these subtests are summed to yield the composite TDI score, which reflects overall olfactory performance [33].

In the threshold subtest, participants were blindfolded and presented with triplets of pens, two containing only the solvent and one containing the target odorant (n-butanol) at varying concentrations. Stimulus concentrations were adjusted using a staircase procedure, increasing after incorrect and decreasing after correct responses. The lowest concentration reliably detected was recorded as the threshold score, with lower scores indicating higher thresholds and thus poorer olfactory sensitivity.

The discrimination subtest was likewise performed under blindfolded conditions, using a triangle paradigm. Participants were presented with three pens, two containing the same odor and one a different odor, and were asked to identify the odd one out. Higher scores indicated a greater ability to differentiate between odors.

Finally, the identification subtest evaluated the ability to recognize and correctly label odors. Participants were presented with 16 everyday odors, each accompanied by four written response alternatives, and asked to select the appropriate one [34].

For children under 12 years, a modified version of the test was applied: instead of the standard three-alternative forced-choice (3AFC) procedure, a two-alternative forced-choice (2AFC) format was used for threshold and discrimination, while the identification subtest included 12 odors matched to pictures rather than words. For children aged 12 years and older, test version allocation (2AFC vs. 3AFC) was determined individually according to developmental stage under medical supervision. Reference values for the 2AFC version were derived from Gellrich et al. [34] for threshold and discrimination and from Lohrer et al. [35] for identification, whereas reference values for the 3AFC version in adolescents (12–17 years) were obtained from Oleszkiewicz et al. [36]. Notably, composite TDI reference values are not available for the 2AFC version.

Based on published reference values, participants’ olfactory abilities were classified as normosmia (10th − 90th percentile), hyposmia (< 10th percentile), or hyperosmia (> 90th percentile) [33, 37].

#### Self-assessment

At T1, children self-reported their perceived ability to smell as good, normal, or poor. At T2, they were also asked whether they noticed any changes in their smell sensitivity and whether it had improved or worsened.

#### Data items and statistics

Data were analyzed using IBM SPSS Statistics version 21 (SPSS Inc., Chicago, IL, USA). Olfactory test scores were analyzed using parametric tests when normally distributed, and non-parametric alternatives when distributional assumptions were not met. A two-sided significance level of *p* < .05 was applied throughout.

Group differences between OBE at T1 and published reference data were examined using unpaired t-tests; when the assumption of equal variances was violated, Welch’s correction was applied. Given that available normative datasets are age- and test-format dependent, participants were stratified into three groups: (i) children < 12 years assessed with the 2AFC paradigm, (ii) children ≥ 12 years assessed with the 2AFC paradigm, and (iii) adolescents ≥ 12 years assessed with the standard 3AFC version. Reference values were applied only at T1, whereas T2 reflects intervention effects and was therefore analyzed independently of normative datasets.

Changes in olfactory outcomes between T1 and T2 were analyzed using paired t-tests. To examine whether the magnitude of anthropometric change was associated with olfactory function changes, Spearman correlation analyses were conducted for change scores (ΔT2–T1). To further explore these associations while controlling for age, sex, and pubertal stage, ANCOVAs were conducted with ΔBMI z-score and Δ% body fat as covariates in separate models, with sex and pubertal stage included as between-subject factors.

Self-assessments of olfactory function were recoded into ordinal variables (T1: impaired, normal, or good; T2: worsened, unchanged, or improved). To examine the association between perceived changes in olfactory ability and objective olfactory performance, Spearman correlation analyses were performed between the T2 self-assessment variable and change scores (ΔT2–T1) for TDI and subscores, as this non-parametric approach accounts for the ordinal scale of the self-assessment variable.

## Results

### Study population

The characteristics of the study population are summarized in Table 1. At T1 (first study assessment), data were available for 59 OBE. Of these, 21 of 31 girls (68%) had reached menarche, and 10 of 28 boys (36%) reported voice changes indicative of puberty. Pubertal status according to Tanner staging was distributed as follows: 12 participants (20%) were in early puberty (stage 2), 33 (56%) in mid-puberty (stages 3–4), and 9 (15%) in late puberty (stage 5). At T2 (pre-discharge assessment), data were available for 50 participants. Between T1 and T2, participants showed a mean weight reduction of 4.4 ± 2.5 kg, equivalent to a decrease of 0.21 ± 0.12 BMI z-scores (*p* < .001).

**Table 1:** Characteristics of study participants

	OBE T1 (*n* = 59)	OBE T2 (*n* = 50)	FDR OBE T1 vs. T2
**Sex** **(****:****)**	28:31	22:28	*n*.s.
**Age (years)**	13.0 ± 1.9	13.0 ± 1.9	n.s.
[Min-Max]	[9–17]	[9–17]	
**BMI z-score**	2.5 ± 0.5	2.4 ± 0.6	< 0.001*
[Min-Max]	[1.3–3.7]	[1.2–3.6]	
**Body fat %**	34.1 ± 5.9^a^	30.8 ± 4.3^b^	< 0.001*
[Min-Max]	[17–46]	[19–40]	

*Notes*: Characteristics of children and adolescents with obesity (OBE) at T1 (first study assessment) and T2 (pre-discharge assessment) are presented as mean ± SD. P values were adjusted for multiple testing using the false discovery rate (FDR). Significance: FDR < 0.05 (*); n.s., not significant. ᵃn = 58; ᵇn = 48

### Enhanced odor discrimination and increased prevalence of hyposmia in identification in younger OBE, with predominantly normosmic performance in older groups

**Fig. 2:**
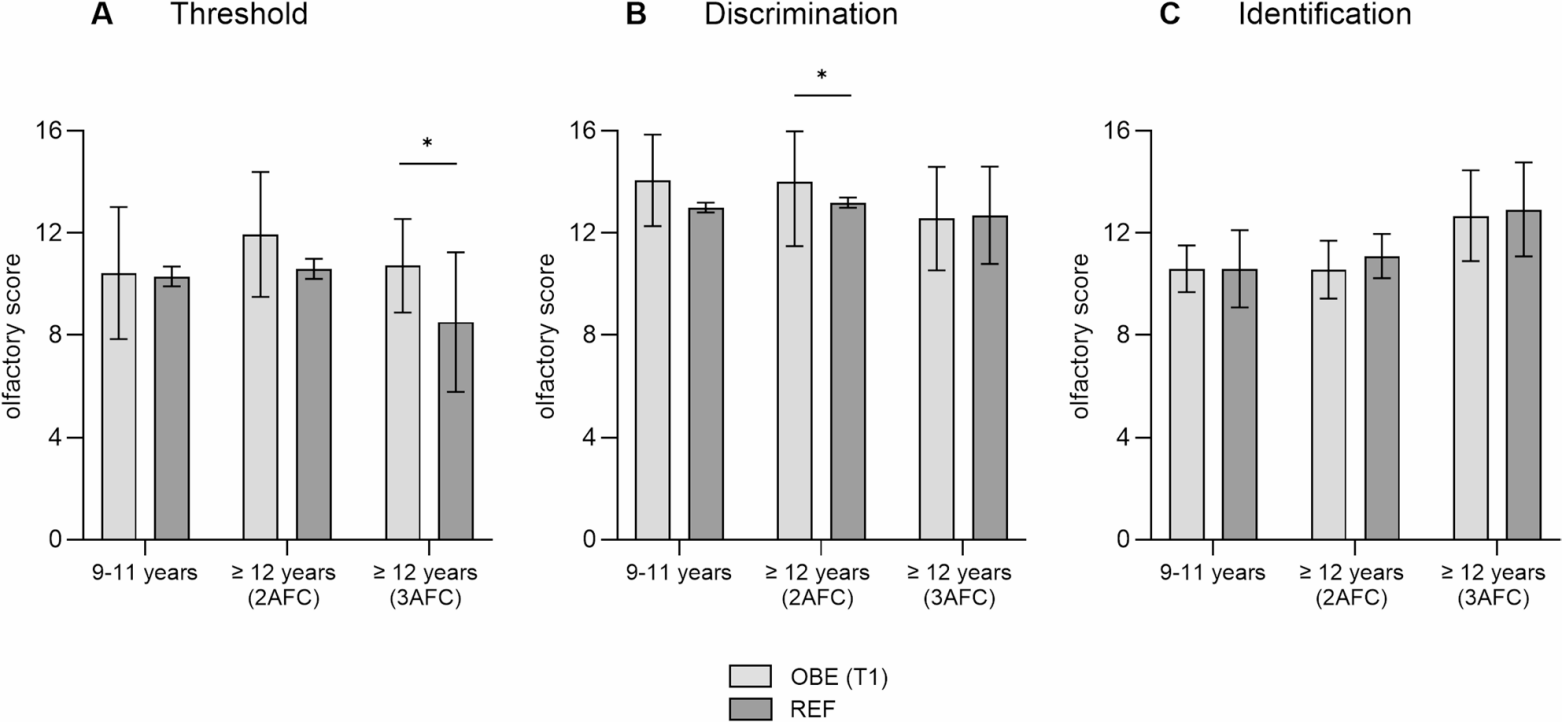
Olfactory function in children and adolescents with obesity in comparison to reference data. Olfactory function in children and adolescents with obesity (OBE) at T1 compared to reference data (REF). Subscales: (A) Threshold, (B) Discrimination, (C) Identification. Higher test scores indicate better performance, with 16 being the maximum. The figure presents OBE group means ± SD with corresponding reference values. Age groups were matched to the available reference datasets and applied test formats (2AFC or 3AFC): for 9–11 years, Gellrich et al. (2017; *n* = 29 for Threshold and Discrimination) and Lohrer et al. (2024; *n* = 169 for Identification); for ≥ 12 years (2AFC), Gellrich et al. (2017; *n* = 30 for Threshold and Discrimination) and Lohrer et al. (2024; *n* = 160 for Identification); for ≥ 12 years (3AFC), Oleszkiewicz et al. (2019; *n* = 1750). Significance of OBE–REF comparisons is indicated as * *p* < .05, ** *p* < .01, *** *p* < .001

OBE younger than 12 years (*n* = 15) exhibited significantly higher odor discrimination scores compared to published normative data for healthy children [34] (*p* = .037), whereas no significant differences emerged in odor threshold or identification (Fig. 2). In OBE aged 12 years and older who completed the full test (*n* = 37) performance was significantly better than normative data for odor threshold (*p* < .001) and the overall TDI score (*p* = .005) [36]. By contrast, OBE aged 12 years and older who completed the shortened test version (*n* = 7), no subscores differed significantly from the normative data, indicating olfactory performance within the expected range [34, 35]. Descriptive statistics for all subscores are provided in Additional file 1: Table 1.

In line with the score-based results, younger OBE (9–11 years) were most frequently classified as hyperosmic in discrimination (60%, 9/15; Table 2). At the same time, 80% (12/15) were categorized as hyposmic in identification, a discrepancy not apparent from the mean scores. Consistent with these subgroup findings, correlation analyses across the full sample confirmed that younger age was associated with higher discrimination (*Spearman r* = − .413, *p* = .001) but poorer identification (*Spearman r* = .465, *p* < .001). Among OBE aged 12 years and older who completed the shortened 2AFC version, classifications indicated predominantly normosmic performance, with 71% (5/7) normosmic and 29% (2/7) hyperosmic in threshold, a more heterogeneous distribution in discrimination, and a predominance of hyposmia (57%, 4/7) in identification. By contrast, in OBE aged 12 years and older assessed with the full 3AFC test, most participants were classified as normosmic across subscores, despite markedly superior mean scores in threshold and TDI, which were not fully reflected in the categorical classification.

**Table 2:** Classification of olfactory function in children and adolescents with obesity at T1: Normosmia (10th–90th percentile), Hyposmia (< 10th percentile), and Hyperosmia (> 90th percentile)

	Hyposmia%	Normosmia%	Hyperosmia%	Literature Cut-off Values of the 10th /90th percentile
**Group 9–11 years** (***n* = 15)**				
Threshold	0	67	33	5.3/15.025¹
Discrimination	13	27	60	11/15¹
Identification	80	20	0	8.8/12.0²
TDI Score	n.a.	n.a.	n.a.	n.a.
**Group ≥ 12 years (2AFC)** (*n* = 7)				
Threshold	0	71	29	7.85/13.75¹
Discrimination	14	43	43	10/15¹
Identification	57	29	14	10/12²
TDI Score	n.a.	n.a.	n.a.	n.a.
**Group ≥ 12 years (3AFC)** (*n* = 37)				
Threshold	3	62	35	5.5/12^3^
Discrimination	22	62	16	10/15^3^
Identification	24	62	14	10/15^3^
TDI Score	5	81	14	28.5/39.25^3^

*Notes*: The table reports the proportions of children in each age group (9–11 years; ≥ 12 years, 2-AFC; ≥ 12 years, 3-AFC) who, according to reference data, fall into the normosmic, hyposmic, or hyperosmic range for each subtest. Reference values are derived from ¹Gellrich et al. (2017), ²Lohrer et al. (2024), and ³Oleszkiewicz et al. (2019) [T: *n* = 802, D: *n* = 547, I: *n* = 1405, TDI: *n* = 384]. n.a. = not available

### Olfactory functions remained largely stable during weight-loss

**Fig. 3:**
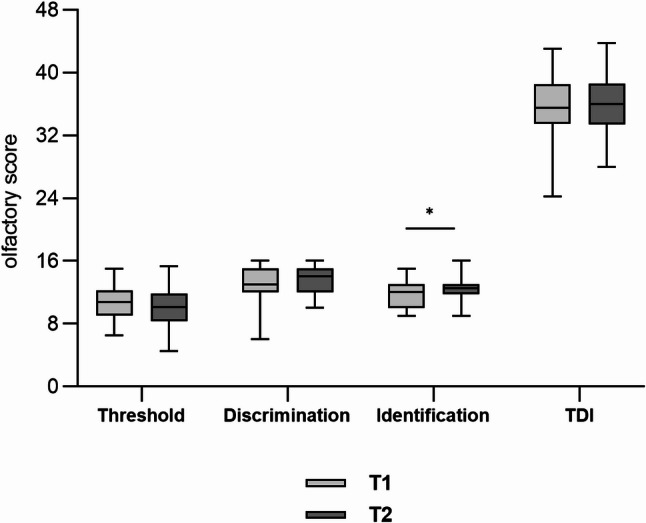
Olfactory function in children and adolescents with obesity between T1 and T2. Olfactory function in children and adolescents with obesity (OBE) at T1 (first study assessment) versus T2 (pre-discharge assessment): subscales Threshold (T), Discrimination (D), and Identification (I). Two-sided t-tests were used. Significance: * *p* < .05. n.a. = not available

Between T1 and T2, identification improved significantly (T1: 11.9 ± 1.9; T2: 12.5 ± 1.4; *p* = .003, d = 0.45), while discrimination (T1: 13.4 ± 1.9; T2: 13.7 ± 1.9), threshold (T1: 10.8 ± 2.3; T2: 10.0 ± 2.6), and TDI (T1: 36.1 ± 3.8; T2: 36.2 ± 3.6) remained largely stable (all *p* > .05, all d ≤ 0.28; Fig. 3, Additional file 1: Table 2).

Despite moderate but significant cross-sectional associations at T1 with age and anthropometrics (body weight, body fat), analyses of change scores revealed no significant associations between changes in olfactory function and anthropometric measures (*Spearman*, all *p* > .05).

To further examine whether the magnitude of anthropometric change was associated with olfactory function changes, ANCOVAs were conducted with ΔBMI z-score and Δ% body fat as covariates separately, controlling for age, sex, and pubertal stage. Neither the magnitude of BMI z-score reduction nor the magnitude of body fat reduction was significantly associated with changes in TDI, odor threshold, or odor identification (all *p* > .05, all η² ≤ 0.081). For odor discrimination, a significant effect of the magnitude of Δ% body fat emerged after adjustment (F(1,36) = 5.18, *p* = .029, η² = 0.126, B = 0.26), with greater body fat loss associated with lower discrimination change scores; this effect was not apparent for ΔBMI z-score (*p* = .913, η² < 0.001).

### Predominantly stable self-reported olfactory function without correlation to objective performance

**Fig. 4:**
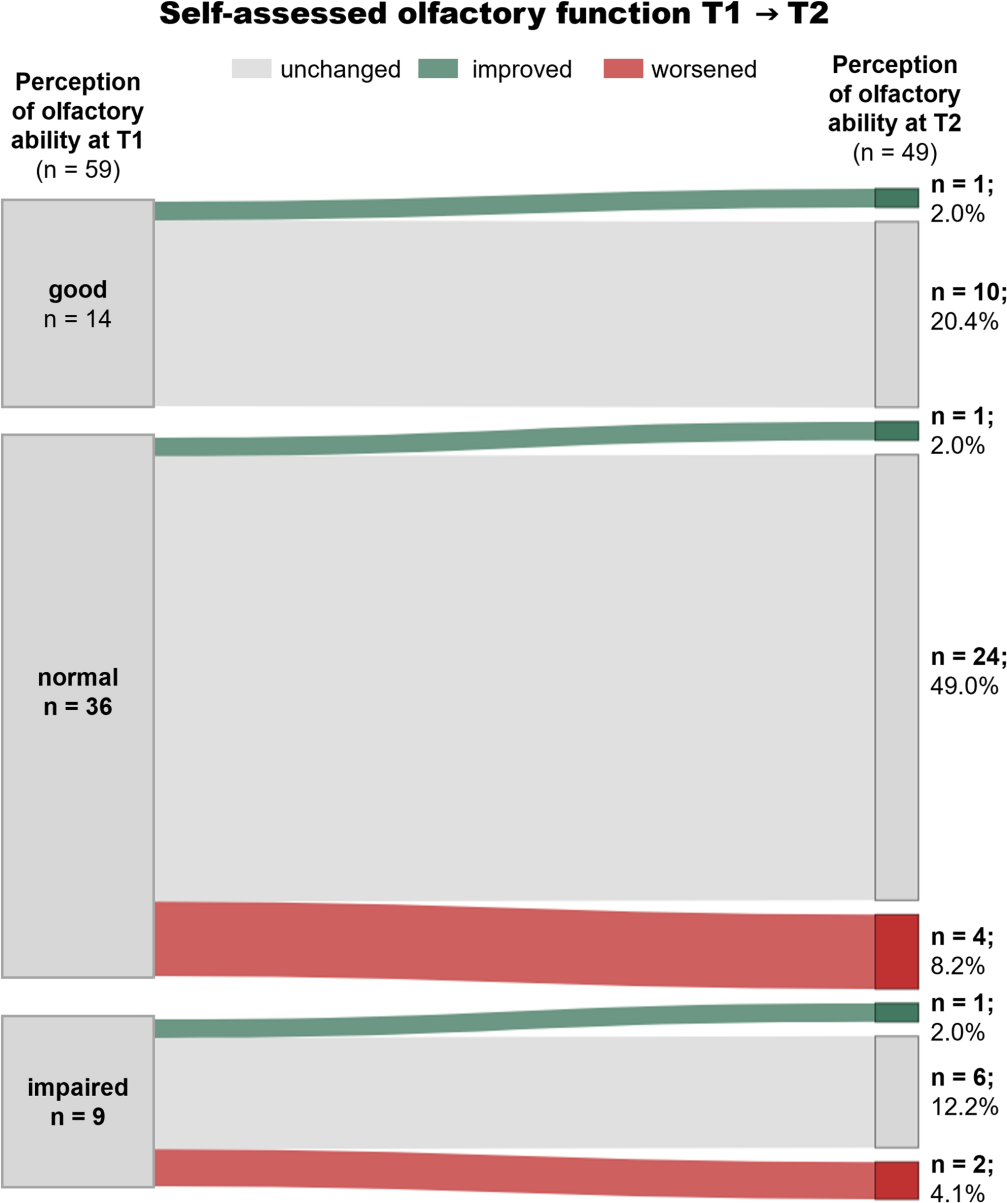
Changes in self-assessed olfactory function from T1 to T2. Flows represent transitions between perceived olfactory categories (enhanced, normal, reduced) from baseline (T1) to follow-up (T2). The width of each flow is proportional to the number of participants (absolute number of participants is indicated). Grey flows indicate stable perception, green flows indicate improvement, and red flows indicate worsening

At T1, 59 children with obesity rated their sense of smell: 36 (61%) reported it as normal, 9 (15%) as diminished, and 14 (24%) as above normal. At T2, 49 children were asked whether they had noticed any change in their sense of smell since T1; 40 (82%) reported no change, 3 (6%) an improvement, and 6 (12%) a decline (Fig. 4). Self-assessed olfactory ability at T1 showed no significant association with objective olfactory performance (TDI: *Spearman* *r* = .097, *p* = .465; threshold: *Spearman* *r* = − .106, *p* = .422; discrimination: *Spearman* *r* = .313, *p* = .016; identification: *Spearman* *r* = − .050, *p* = .706). Perceived changes in olfactory ability at T2 showed no significant association with objective change scores (ΔT2–T1) for TDI or any subscore (TDI: *Spearman* *r* = .112, *p* = .459; threshold: *Spearman* *r* = .131, *p* = .384; discrimination: *Spearman* *r* = .049, *p* = .744; identification: *Spearman* *r* = .067, *p* = .658).

## Discussion

The present study provides a differentiated view of olfactory function in children and adolescents with obesity. Overall, olfactory performance was largely within the normative range when compared with reference data [34–36]. However, an age- and domain-specific pattern emerged, with younger participants showing enhanced odor discrimination alongside a higher prevalence of hyposmia in identification, whereas older participants exhibited predominantly normosmic performance and higher odor threshold scores. During short-term inpatient weight loss, olfactory function remained largely stable. Although odor identification improved modestly over time, the remaining olfactory measures did not change significantly, and changes in olfactory performance were not associated with the magnitude of weight loss.

Importantly, these findings indicate a heterogeneous and domain-specific profile of olfactory function across age groups, characterized by the coexistence of impairments and areas of relatively enhanced performance. This pattern may partly reflect developmental influences. Cognitive processes such as semantic knowledge, memory, and familiarity with odor descriptors, which are known to influence odor identification [38, 39], are still maturing during childhood and adolescence. This may help explain the divergence between relatively strong discrimination performance and lower identification scores in younger participants [40, 41], as odor identification relies more strongly on memory, semantic knowledge, and verbal labeling than other olfactory tasks [42]. In addition, higher odor threshold scores in older participants may reflect differences in perceptual processing or more consistent task performance, but may also be influenced by methodological factors and variability inherent to threshold measurements [42]. These findings suggest that age-related factors may contribute to variability in olfactory function, although the relative contribution of age versus weight-related influences cannot be determined from the present data.

However, whether such variability has functional relevance for eating behavior and, consequently, body weight remains unclear. Differences in olfactory sensitivity may affect how food-related odor cues are perceived and processed and could thereby contribute to variability in eating behavior, as food-related sensory cues, including olfactory cues, have been shown to modulate appetite, food preferences, though their impact on actual intake may be more limited [9, 43, 44]. In contrast, a recent study reported increased emotional eating and reduced interoceptive accuracy in children with overweight or obesity, despite comparable overall olfactory performance to normal-weight controls [19], suggesting that alterations in eating behavior may occur independently of global olfactory function. Taken together, these findings support a differentiated perspective in which olfactory function may act as a modulatory rather than a primary determinant of obesity-related eating behavior, potentially influencing how individuals respond to dietary interventions and adhere to treatment recommendations.

When considered in a broader lifespan context, the present findings differ from patterns reported in adult populations, where reduced olfactory function with increasing body weight has been described, albeit inconsistently [14, 45]. One potential explanation is that children and adolescents typically have a shorter duration of exposure to obesity-related metabolic alterations, which may limit cumulative effects on olfactory pathways, although direct evidence for this assumption remains limited [12, 46]. This suggests that, if such a relationship exists in younger populations, it may be more strongly influenced by developmental and contextual factors than in adults [41], though direct comparisons with healthy-weight peers are needed to confirm this.

Another key finding is the limited change in olfactory function during short-term weight loss. Apart from a modest increase in odor identification, olfactory performance remained largely stable. However, when controlling for age, sex, and pubertal stage, a significant association between the magnitude of body fat reduction and changes in odor discrimination was observed. Since discrimination scores at T1 exceeded age-matched normative values, the subsequent decline may represent a regression towards the reference range rather than a functionally relevant impairment. While some studies in adults, particularly in bariatric populations, have reported improvements in olfactory sensitivity following weight loss, the present findings do not support a comparable effect in pediatric obesity. This may indicate that olfactory function represents a relatively stable individual characteristic over short time frames or that the approximately 27-day observation window and modest weight loss achieved in the present study fall below the threshold necessary for measurable olfactory adaptation. Studies reporting olfactory improvements following bariatric surgery typically assessed outcomes after six months or more [14, 15], suggesting that more sustained or pronounced weight loss may be required. In addition, interindividual variability in olfactory domains may have contributed to the absence of clear associations at the group level.

Self-reported olfactory ability showed only limited correspondence with objective test performance and did not reflect changes over time. This is consistent with previous findings reporting low agreement between subjective and psychophysical measures of chemosensory function [47, 48]. One possible explanation is that self-assessments in untrained individuals are influenced by factors such as nasal airflow or limited awareness of olfactory perception in daily life, leading to underrecognition of subtle impairments [47]. In line with this, evidence from the COVID-19 pandemic suggests that self-reports become more reliable when impairments are pronounced or occur suddenly [49, 50]. However, pediatric data on the validity of self-reported olfactory function remain limited, and developmental factors such as limited odor knowledge may further complicate self-assessment [41, 42], potentially reducing the reliability of self-reports in children and adolescents. From a clinical perspective, this suggests that self-report measures should be interpreted cautiously and ideally complemented by standardized psychophysical testing, particularly in pediatric settings.

Beyond this, these findings indicate that changes in anthropometric measures alone may not necessarily be accompanied by measurable changes in sensory perception. At the same time, variability across olfactory domains may contribute to interindividual differences in how children experience food environments and respond to dietary interventions, potentially influencing adherence and long-term weight management. In line with current approaches to weight management [51, 52], which integrate dietary, behavioral, and environmental components, future strategies may benefit from considering individual responses to food-related cues, particularly with regard to sustaining behavioral change. While the present data do not allow conclusions regarding behavioral or clinical outcomes, such sensory differences may be relevant for understanding variability in intervention response. Future studies should therefore examine whether these sensory characteristics are associated with long-term weight loss maintenance, adherence to lifestyle interventions, and sustained treatment success.

### Strengths and limitations

The present study has several strengths that support the interpretation of these findings. It offers rare longitudinal data on olfactory function in pediatric obesity, particularly in a clinical setting. The inpatient rehabilitation context enabled a structured and standardized assessment environment, reducing external variability and ensuring consistent implementation of the intervention. Methodologically, the study differentiates between multiple olfactory domains, including threshold, discrimination, identification, and composite TDI performance, rather than relying on a single summary measure. It further combines objective psychophysical testing with self-reported assessment and interprets findings in relation to age-specific normative data. Taken together, these features enable a comprehensive and nuanced characterization of olfactory function in children and adolescents with obesity.

However, several limitations should be considered when interpreting these findings. First, the absence of a normal-weight control group limits conclusions regarding the influence of weight status per se on olfactory function, and differences in sampling context between published normative datasets and the present sample cannot be fully excluded. Second, different test versions were used depending on age and developmental stage, which was necessary for clinical feasibility but limits direct comparability across subgroups. In this context, it should be noted that the 2AFC version of the test is generally considered less sensitive and less reliable than the standard 3AFC procedure [34], which may have influenced the results, particularly in younger participants. Third, subgroup sizes, particularly in the older 2AFC group, were small, reducing statistical power and increasing uncertainty in categorical classifications; the same limitation applies to the ANCOVA models, where small subgroup sizes increase the risk of model overfitting. Fourth, T1 was assessed approximately 7 days after admission, precluding a strictly pre-intervention baseline, though substantial intervention effects within this timeframe are unlikely. Finally, the relatively short intervention period may have limited the detection of longer-term olfactory adaptations.

## Conclusions

In conclusion, the present findings suggest that olfactory function in children and adolescents with obesity is largely within the normative range and is not characterized by a generalized impairment but rather by an age-dependent, domain-specific pattern. Olfactory function appears largely stable during short-term inpatient weight loss. At the same time, variability across olfactory domains may still be behaviorally relevant, as it could influence how children perceive and respond to food-related cues, with potential implications for dietary behavior, intervention adherence, and long-term weight management. Future studies with larger samples, longer follow-up periods, normal-weight control groups, and harmonized testing approaches are needed to further clarify the role of olfaction in long-term weight management outcomes.

## Supplementary Information

Below is the link to the electronic supplementary material.

Supplementary Material 1: Additional File 1: Table 1–Table 2. Table 1: Olfactory function in children and adolescents with obesity in comparison to reference data. Table 2: Olfactory function of children & adolescents with obesity upon weight-loss.

## Data Availability

The data that support the findings of this study are available from the corresponding author, BK, upon reasonable request.

## Abbreviations

2AFC: Two-alternative forced-choice
3AFC: Three-alternative forced-choice
BMI: Body mass index
DROMLIN: PreDictor Research in Obesity during Medical Care Weight Loss in Children and Adolescents during Inpatient Rehabilitation
OBE: Children and adolecents with obesity
REF: Reference data
TDI: Threshold-Discrimination-Identification
WHO: World Health Organization
