# Supplementary material for: Changes in olfactory function in children and adolescents with obesity following an inpatient weight loss program: a longitudinal study
Source: BMC Med. 2026 Jun 17;24:361. doi: 10.1186/s12916-026-05007-3 (PMC13277082; doi:10.1186/s12916-026-05007-3)
Supplement: Supplementary file 1 — Supplementary Material 1: Additional File 1: Table 1–Table 2. Table 1: Olfactory function in children and adolescents with obesity in comparison to reference data. Table 2: Olfactory function of children & adolescents with obesity upon weight-loss. [file 12916_2026_5007_MOESM1_ESM.docx]

**Changes in olfactory function in children and adolescents with obesity following an inpatient weight loss program: a longitudinal study – Additional file**

Bea Klos^1*^, Helene Sauer^1^, Verena Steinhauser^1^, Jessica Godwin^1^, Kathrin Ohla^2^, Valentin A. Schriever^3^, Stephan Zipfel^1^, Paul Enck^1^ and Isabelle Mack^1^

^1^Department of Psychosomatic Medicine and Psychotherapy, University Medical Hospital Tübingen, Germany

^2^Science & Research, dsm-firmenich, Satigny, Switzerland

^3^Goethe University Frankfurt, Department of Pediatrics, Division of Pediatric Neurology, Neurometabolics and Prevention, Frankfurt (Main), Germany

**Correspondence:** Bea Klos, [bea.klos@med.uni-tuebingen.de](mailto:bea.klos@med.uni-tuebingen.de), +49 7071 29 85614

**Table 1: Olfactory function in children and adolescents with obesity in comparison to reference data.**

|  | **OBE T1** | **REF** | **Statistics** |  |
| --- | --- | --- | --- | --- |
| **Group 9-11 years** | ***n* = 15** | **¹*n* = 29; ²*n* = 169** | **p-value** | **Intermediate values** |
| Threshold | 10.4 ± 2.6 | 10.3 ± 0.4¹ | .845 | t(14.43) = 0.199 |
| Discrimination | 14.1 ± 1.8 | 13.0 ± 0.2¹ | **.037*** | t(14.24) = 2.296 |
| Identification | 10.6 ± 0.9 | 10.6 ± 1.5² | >.999 | t(21.57) = 0.000 |
| TDI score | 35.1 ± 3.9 | n.a. | n.a. | n.a. |
| **Group ≥ 12 years (2AFC)** | ***n* = 7** | **¹*n* = 30; ²*n* = 160** | **p-value** | **Intermediate values** |
| Threshold | 11.9 ± 2.4 | 10.6 ± 0.4¹ | .197 | t(6.07) = 1.447 |
| Discrimination | 14.0 ± 2.5 | 13.2 ± 0.2¹ | .433 | t(6.02) = 0.840 |
| Identification | 10.6 ± 1.1 | 11.1 ± 0.9² | .122 | t(165) = 1.155 |
| TDI score | 36.5 ± 4.7 | n.a. | n.a. | n.a. |
| **Group ≥ 12 years (3AFC)** | ***n* = 37** | **^3^*n* = 1750** | **p-value** | **Intermediate values** |
| Threshold | 10.7 ± 1.8 | 8.5 ± 2.7 | **<.001***** | t(43.72) = 6.963 |
| Discrimination | 12.6 ± 2.0 | 12.7 ± 1.9 | 0.706 | t(582) = 0.378 |
| Identification | 12.7 ± 1.8 | 12.9 ± 1.8 | 0.425 | t(1440) = 0.798 |
| TDI score | 36.0 ± 3.5 | 34.0 ± 4.2 | **0.005**** | t(419) = 2.776 |

Olfactory function in children and adolescents with obesity (OBE) at T1 versus reference data: subscales Threshold (T), Discrimination (D), Identification (I), and composite TDI. The table reports OBE means stratified by age (9–11, 12, 13–17 years, according to which test they used), corresponding reference values (¹Gellrich et al., 2017; ²Lohrer et al., 2024; ^3^Oleszkiewicz et al., 2019 (T: *n* = 802, D: *n* = 547, I: *n* = 1405, TDI: *n* = 384)), p-values for OBE–REF comparisons, and test statistics (*t*(df), SED). Two-sided t-tests were used, with Welch adjustment when the F-test indicated unequal variances. Significance: * *p*<0.05, ** *p*<0.01, *** *p*<0.001. n.a. = not available.

**Table 2: Olfactory function of children & adolescents with obesity upon weight-loss.**

|  | **T1** (Mean ± SD)  [Min–Max] | **T2**  (Mean ± SD)  [Min–Max] | **n**  (paires) | **t** | **df** | **p-value** | **Cohen’s d** |
| --- | --- | --- | --- | --- | --- | --- | --- |
| **Threshold** | 10.8 ± 2.1 | 10.0 ± 2.6 | 50 | 49 | 1.937 | .053 | 0.28 |
|  | [6.5-15.0] | [4.5-15.3] |  |  |  |  |  |
| **Discrimination** | 13.4 ± 1.9 | 13.7 ± 1.9 | 50 | 49 | 1.498 | .141 | 0.21 |
|  | [6.0-16.0] | [10.0-16.0] |  |  |  |  |  |
| **Identification** | 11.9 ± 1.9 | 12.5 ± 1.4 | 50 | 49 | 3.167 | **.003**** | 0.45 |
|  | [9.0-15.0] | [9.0-16.0] |  |  |  |  |  |
| **TDI score** | 36.1 ± 3.8 | 36.2 ± 3.6 | 50 | 49 | 0.191 | .850 | 0.03 |
|  | [24.3-43.0] | [28.0-43.8] |  |  |  |  |  |

Olfactory function in children and adolescents with obesity (OBE) at T1 versus at T2: subscales Threshold (T), Discrimination (D), Identification (I), and composite TDI. Data presented as Mean ± SD with Min and Max (the higher the respective value, the more sensitive the subjects Olfaction). Two-sided paired t-tests were used; df = 49 for all comparisons. Effect sizes are reported as Cohen's d, calculated as the mean difference divided by the standard deviation of differences. Significance: * p<0.05, ** p<0.01, *** p<0.001.
